# What Matters for Depression and Anxiety During the COVID-19 Quarantine?: Results of an Online Cross-Sectional Survey in Seoul, South Korea

**DOI:** 10.3389/fpsyt.2022.706436

**Published:** 2022-03-07

**Authors:** Hye-Young Kwon, Yongjoo Kim, Seung-Young Lee

**Affiliations:** ^1^Division of Biology and Public Health, Mokwon University, Daejeon, South Korea; ^2^Seoul Health Foundation, Seoul, South Korea; ^3^College of Korean Medicine, Sangji University, Wonju, South Korea; ^4^College of Nursing, Kyung Hee University, Seoul, South Korea

**Keywords:** quarantine, depression, anxiety, quality of life, EQ-5D, COVID-19

## Abstract

**Background:**

Amid the COVID-19 pandemic, quarantine measures are key to containing the spread of the virus. Millions of people have been required to quarantine throughout the pandemic; the quarantine itself is considered detrimental to mental health conditions.

**Objective:**

This study aims to investigate the factors associated with depression and anxiety among quarantined people in Seoul, South Korea.

**Methods:**

An online cross-sectional survey was administered from October to November 2020 involving people who were living in Seoul, aged 19 years or above, under a 2-week mandatory quarantine. Their mental health status was measured using the Patient Health Questionnares-9 (PHQ-9) and the General Anxiety Disorder-7 (GAD-7).

**Results:**

Overall, 1,135 respondents were finally included, resulting in a 22.0% response rate. After controlling for potential confounders, variables, such as the “second half of quarantine period” (OR = 1.78 95% CI: 1.10–2.88), “female” (OR = 1.91 95% CI: 1.16–3.16), and “having pre-existing depression” (OR = 8.03 95% CI: 2.96–21.78) were significantly associated with depression while being quarantined. Those with correct knowledge about the rationale behind for the quarantine (OR = 0.39 95% CI: 0.21–0.72), an understanding of quarantine rules (OR = 0.68 95%CI: 0.52–0.91), and those who felt supported by others (OR = 0.74 95% CI: 0.55–0.99) were less likely to develop depression while quarantining. Similarly, anxiety was significantly associated with the second week (OR = 4.18 95% CI: 1.44–12.09), those with an unstable job status (OR = 3.95 95% CI: 1.60–9.79), perceived support (OR = 0.66, 95% CI: 0.45–0.96), and the fear of being infected (OR = 7.22 95% CI: 1.04–49.95).

**Conclusions:**

This study highlights the need to develop precautionary measures to prevent depression and anxiety among people undergoing COVID-19 quarantine. In particular, individuals with depression prior to quarantine should be carefully monitored during the quarantine. Further studies with larger populations are needed.

## Introduction

Owing to the global efforts to overcome the SARS-CoV-2 (COVID-19), widespread vaccinations have finally become a reality (1, 2). Nevertheless, the world has been battling this novel virus since the first case was reported from Wuhan, China in late 2019.

As the pandemic progressed, strategies to have targeted means of identifying cases, minimizing the spread of the virus, and mitigating its clinical manifestations among those already infected (3, 4). Given that COVID-19 is droplet borne, restricting the spread of the virus is key to controlling the pandemic's longevity. Hence, a quarantine strategy-identifying cases through timely but accurate testing so as to determining who to quarantine, and in which way—is vital to containing this virus. Accordingly, more than a billion people across more than 50 countries and territories were asked to remain confined to their homes (5). A growing number of studies demonstrated that health outcomes, notably, quality of life and psychological burden among quarantined people have significantly worsened (6–15). Thus, it is necessary to explore the factors contributing to poor health outcomes during quarantine so as to identify vulnerable groups that may require preemptive interventions. Previous research documented findings stating that sociodemographic features, such as age, gender, education, marital status, prolonged one's quarantine period, and pre-existing morbidities were associated with poor mental health conditions during the quarantine period (7–15).

Known for its successful control over this new virus (16–18), South Korea, armed with knowledge gained from the Middle East Respiratory Syndrome (MERS) epidemic in 2015 (19), has enforced a 14-day mandatory quarantine for inbound travelers and anyone who comes into contact with confirmed cases. This has been achieved via meticulous contact tracing and a widespread and aggressive testing policy in place since the early stages of the outbreak. According to the most recent data (20), South Korea's case fatality rate of COVID-19 was 1.78%, with 1,316 deaths and 73,918 cases (as of January 20, 2021), and the total number of tested people was 5,043,988, approximately 9.8% of the total population. Additionally, the total number of quarantined individuals reached 820,223 (as of November 17, 2020) (21).

However, factors associated with mental disorders among people under mandatory quarantine have not been sufficiently explored in the Korean context. Therefore, this study investigates factors associated with mental health disorders (depression and anxiety) among individuals undergoing COVID-19 quarantine; to the best of our knowledge, this is the first study on this topic pertaining to the context of South Korea.

## Methods

### Subjects

The Seoul COVID-19 Study (SCS) is a joint project initiated by the Seoul Metropolitan Government and the Seoul Health Foundation. It aims to investigate the performances of the Seoul government's countermeasures against COVID-19. The SCS focuses on people who used the screening posts for COVID-19 testing, the asymptomatic cases admitted in the residential centers for surveillance, and quarantined people who tested negative. The SCS for quarantine (SCS-Q) has been conducted from October to November 2020 involving those who live in Seoul, covering those above the age of 19 who were under the 2-week mandatory quarantine at the time of the study.

**Figure 1:**
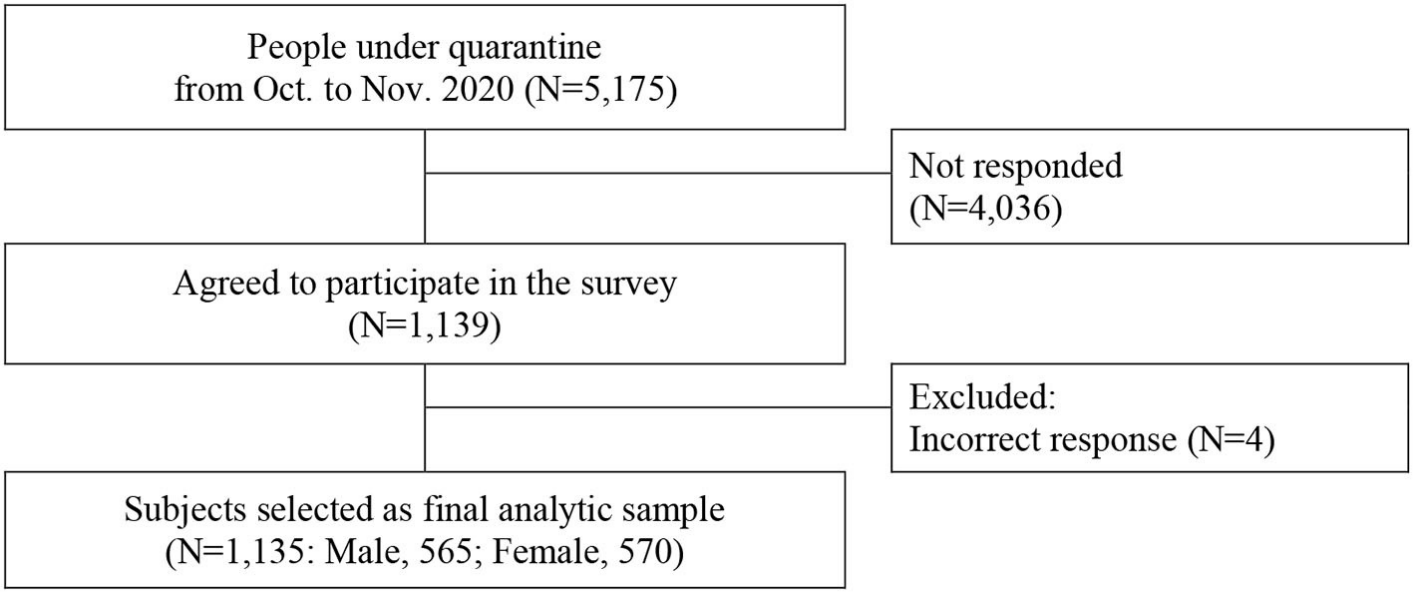
Selection process of the study population.

Considering the legal imposition of the no-contact rule with currently quarantined people, an online cross-sectional survey was designed. The targeted respondents were provided with the information regarding this study via text messages containing the URL of the survey questionnaires, and they voluntarily participated in the current study's cross-sectional online survey. A total of 5,175 people under quarantine were asked to participate. Responding to the survey was based on the participants' consent. Of those asked to participate, 1,139 (22.0% response rate) out of them agreed and filled in the questionnaires. Four individuals' answers were excluded due to incorrect response about quarantined days. Finally, 1,135 respondents were included in this study (Figure 1). Participation was consensual.

### Outcomes

#### Depressive Symptoms

Depressive symptoms among quarantined people were evaluated using the Patient Health Questionnaires-9 (PHQ-9). The PHQ-9 is a validated measure of depressive symptoms and widely used in primary care and research settings. It contains nine items evaluating symptomatology of depression during the previous 2 weeks, including lack of interest, feeling depressed, sleep-related troubles, feeling tired, appetite change, feeling guilty or indulging in self-blame, concentration issues, feeling restless/slowed down, and suicidal ideations (22). The response options for each question were “never,” “several days,” “more than half of the days,” and “almost every day,” scored from 0 to 3 in order, indicating the perceived frequency of depressive symptoms (over the 2 weeks leading up to the questionnaire). The total score ranges from 0 to 27. The higher the score, the more severe the depressive symptoms (22). Prior literature has suggested using a cut-off of 10 to detect major depressive disorder (with a sensitivity 0.85 and a specificity 0.89) (23). Previous research documented the reliability and validity of the Korean translated version of PHQ-9 (24). In this study, we set “during the quarantine” as the timeframe of the PHQ-9 instead of “during the previous 2 weeks.” In this study, the Cronbach's alpha was found to be 0.89.

#### Anxiety Symptoms

The Generalized Anxiety Disorder-7(GAD-7), a widely used instrument, was employed to assess anxiety disorders. It is a seven-item questionnaire regarding the symptomatology of anxiety over the past 2 weeks (leading up to the questionnaire). It includes questions regarding feeling anxious/nervous, uncontrollable worrying, trouble relaxing, feeling annoyed/irritable, and feeling afraid (25). Response options per item indicate the perceived frequency of the anxiety symptom specified for each concern in the 2 weeks leading up to the questionnaire; these are labeled as “never (0),” “several days (1),” “more than half of the days (2),” and “almost every day (3),” with a total score ranging from 0 to 21 (25). Previous studies have reported that GAD-7 as an appropriate assessment tool for detecting generalized anxiety disorder (at 89% sensitivity and 82% specificity when using a cut-off value of 10 or above) when compared to a structured psychiatric interview (26). The reliability and validity of the Korean translated version of the GAD-7 has been previously reported (27). We set “during the quarantine” as the timeframe for the GAD-7 instead of “over the past 2 weeks.” In this study, the Cronbach's alpha was found to be 0.93.

#### Health-Related Quality of Life

A widely used generic instrument of health-related quality of life (HRQoL), the EuroQol-5Dimensions (EQ-5D) (28), was employed to assess HRQoL among the quarantined. The EQ-5D comprises five questions concerning mobility, self-care, usual activities, pain or discomfort, and psychological status. The EQ-5D scores ranging from 0 to 1, indicating death to perfect health, were calculated based on the Korean Tariff (29, 30).

### Covariates

Sociodemographic characteristics of the respondents were collected. Additionally, we drew information regarding their health status before the quarantine by enquiring about any pre-existing chronic diseases including hypertension, diabetes and depression, and asking them to self-rate their health status.

Furthermore, we included variables related to health administration for quarantine. First, it was hypothesized that it is vital for the presence of trust in health authorities—who have taken countermeasures against COVID-19 to adopt sound quarantine, and to correctly understand why people should be confined at home for 2 weeks and under what conditions. Thus, “trust in health authorities,” “reason for being quarantined (e.g., inbound travelers and contacts with a confirmed case),” and “knowledge about the rationale for quarantine (e.g., to protect myself or to protect others)” were assessed on a five-point Likert scale.

For an effective 14-day mandatory quarantine, an understanding of the quarantine rules, the perceived support received during quarantine, and the satisfaction with the essential supplies provided free of charge by local offices were asked on a five-point Likert scale.

Additionally, questions about “quarantine days elapsed at the time of survey” and “concerns, such as fear of infection, financial crisis, and the risk of unemployment” were asked. In particular, number of days of quarantine elapsed at the time of survey was divided into two groups; first week (Day1–Day7) and second week (Day8–Day14).

### Statistical Analysis

Categorical variables were expressed as frequencies and percentages, and continuous variables via their means and standard deviations (SDs). Chi-square test for categorical variables and the Student's *t*-test for means were performed. Mann-Whitney median test was used for the skewed distribution. Fisher's exact test for categorical variables was used when appropriate.

To identify factors contributing to health outcomes during quarantine, multivariable regression models were applied by considering the distribution and attributes of the outcome variables. For example, a standard logistic regression was employed for the dichotomous outcome variables such as depression and anxiety. However, as the EQ-5D scores range from 0 to 1 with a left-skewed distribution, a beta logit regression was used after rescaling the EQ-5D scores to avoid bounded values (31). All statistical analyses were performed using the SAS 9.4 (SAS Institute Inc., Cary, NC).

## Results

### Basic Characteristics of Study Population

Table 1 shows the basic characteristics of the respondents. The average age of respondents was 39.00 (SD 12.54), which is relatively younger than the general population, with a significant difference between male and female (*p* < 0.0001). Most respondents were salaried workers (males 60.0% vs. females 49.8%). However, women constituted a relatively bigger proportion of the economically inactive group, which includes housewives, students, and the unemployed (women 36.1% vs. men 18.6%). Men were more likely to have pre-existing diseases, such as hypertension and diabetes than women (*p* < 0.0001). Self-reported pre-existing depression was more prevalent in women than in men (3.0% vs. 1.4%) at the 10% of significance level (*p* = 0.0722).

**Table 1:** Baseline characteristics of the study population.

**Variables**	**Total**		**Male**		**Female**		**P-value**
	**(*****N*** **=** **1,135)**		**(*****N*** **=** **565)**		**(*****N*** **=** **570)**		
Age, Mean (SD)	39.00	(12.54)	41.00	(12.71)	37.02	(12.07)	<0.0001
Age group							0.0002
19 to 40	607	(53.5%)	269	(47.6%)	338	(59.3%)	
40 to 65	505	(44.5%)	280	(49.6%)	225	(39.5%)	
65 and over	23	(2.0%)	16	(2.8%)	7	(1.2%)	
Marital status							0.0009
Married	588	(51.8%)	320	(56.6%)	268	(47.0%)	
Single	492	(43.3%)	227	(40.2%)	265	(46.5%)	
Divorced/Widowed	55	(4.8%)	18	(3.2%)	37	(6.5%)	
Income							NS
Lowest	138	(12.2%)	70	(12.4%)	68	(11.9%)	
Middle-low	473	(41.7%)	224	(39.6%)	249	(43.7%)	
Middle-high	484	(42.6%)	251	(44.4%)	233	(40.9%)	
Highest	40	(3.5%)	20	(3.5%)	20	(3.5%)	
Employment status							<0.0001
Salaried workers	623	(54.9%)	339	(60.0%)	284	(49.8%)	
Employer/Self-employed	98	(8.6%)	65	(11.5%)	33	(5.8%)	
Economically inactive^*^	311	(27.4%)	105	(18.6%)	206	(36.1%)	
Others	103	(9.1%)	56	(9.9%)	47	(8.2%)	
Education							NS
High school or Less	254	(22.4%)	116	(20.5%)	138	(24.2%)	
Tertiary education	881	(77.6%)	449	(79.5%)	432	(75.8%)	
Family size							NS
Living alone	192	(16.9%)	105	(18.6%)	87	(15.3%)	
Others	943	(83.1%)	460	(81.4%)	483	(84.7%)	
Self-reported predisposing diseases							
Yes	201	(17.7%)	127	(22.5%)	74	(13.0%)	<0.0001
- Hypertension	96	(8.5%)	76	(13.5%)	20	(3.5%)	<0.0001
- Diabetes	51	(4.5%)	40	(7.1%)	11	(1.9%)	<0.0001
- Depression	25	(2.2%)	8	(1.4%)	17	(3.0%)	0.0722
Self-ranked health (Likert = 5)	3.54	(0.77)	3.56	(0.78)	3.52	(0.76)	NS

* *Economically inactive group included students, housewives and the unemployed*.

### Quarantining

Table 2 presents the general information regarding quarantining as determined via a five-point Likert scale; these items include quarantine days at the time of survey, quarantine-related knowledge and understanding, social support, essential supplies provided by district offices, and trust in health authorities among the respondents under quarantine. The mean quarantine period at the time of survey response was 6.52 (SD 3.98) days; 68.6% of the respondents were quarantined owing to the contact with confirmed cases (men 62.7% vs. women 74.6%, *p* < 0.0001); the remaining participants were inbound travelers. Most quarantined people correctly understood that quarantine is necessary to protect others (men 91.7% vs. women 91.4%) and showed a high degree of confidence in the health authorities (4.09 SD 0.93 out of 5.0) who have been planning and implementing countermeasures (including quarantine) against the pandemic (men 4.12 SD 0.93 vs. women 4.06 SD 0.93). The difference in understanding of the quarantine instructions was marginally significant between men and women (4.49 [SD 0.70] vs. 4.40 [SD 0.72], respectively, *p* = 0.045); the overall score was high at 4.44 (SD 0.71). Perceived support during quarantine scored 3.73(SD 1.06) with no significant difference in men (3.71 SD 0.87) and women (3.76 SD 0.92). The respondents were mostly satisfied with the quarantine supplies being provided by the district public health centers (3.58 SD 1.28).

**Table 2:** Characteristics of quarantine (Frequency & 5-point Likert scale).

**Variables**	**Total**		**Male**		**Female**		**P-value**
	**(*****N*** **=** **1,135)**		**(*****N*** **=** **565)**		**(*****N*** **=** **570)**		
Quarantine days, Mean (SD)	6.52	(3.98)	6.51	(3.85)	6.54	(4.10)	NS
Reason for quarantine, *N* (%)							
Contacts traced	779	(68.6%)	354	(62.7%)	425	(74.6%)	<0.0001
Inbound travelers	356	(31.4%)	211	(37.3%)	145	(25.4%)	
Knowledge on quarantine, *N* (%)							
To protect others	1,039	(91.5%)	518	(91.7%)	521	(91.4%)	NS
To protect myself	44	(3.9%)	20	(2.7%)	24	(4.2%)	
Don't know	35	(3.1%)	22	(2.9%)	13	(2.3%)	
Others	17	(1.5%)	5	(0.7%)	12	(2.1%)	
Trust in Health Authorities, Mean (SD)	4.09	(0.93)	4.12	(0.93)	4.06	(0.93)	NS
Understanding the instructions, Mean (SD)	4.44	(0.71)	4.49	(0.70)	4.40	(0.72)	0.0449
Perceived support during quarantine, Mean (SD)	3.73	(1.06)	3.71	(0.87)	3.76	(0.92)	NS
Essential supplies, Mean (SD)	3.58	(1.28)	3.57	(1.32)	3.59	(1.25)	NS

### Outcomes

**Figure 2:**
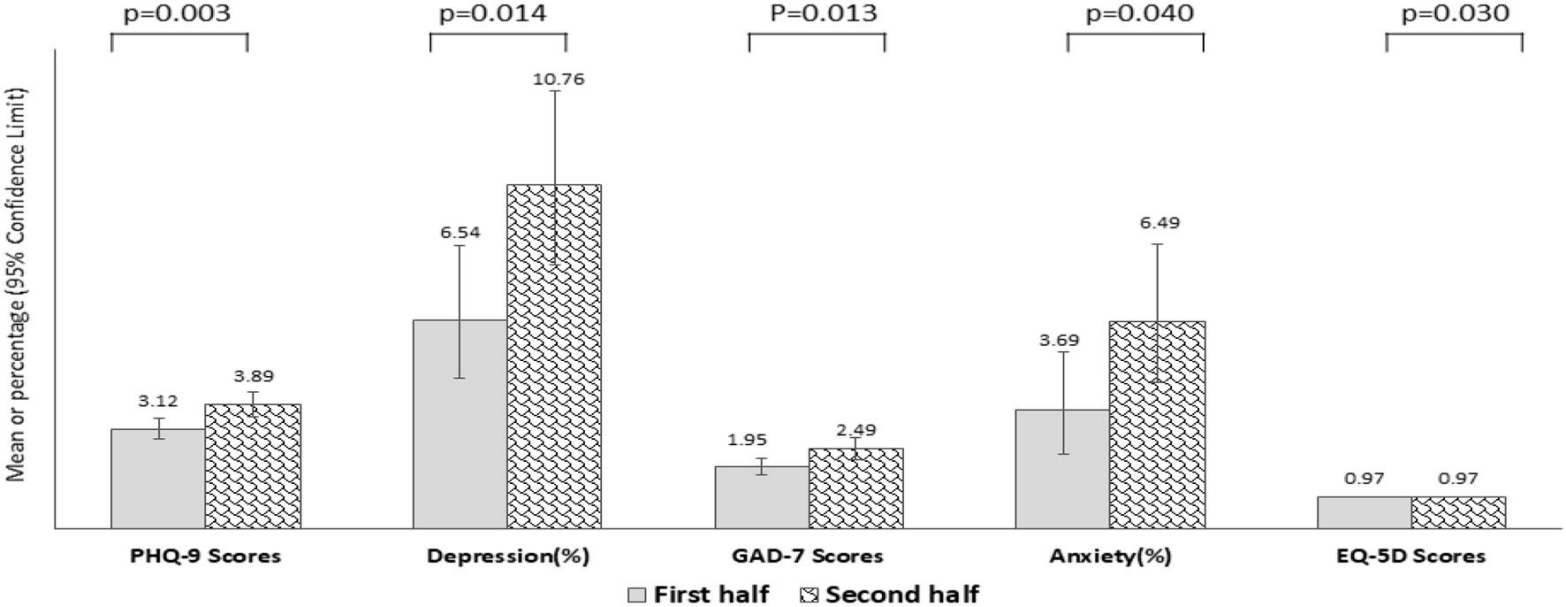
Depression, Anxiety, and Health-related Quality of life by quarantine period (First vs. Second week during the quarantine).

The differences in depression and anxiety as per the quarantine period was divided into the first week (Day1–Day7) and the second week (Day8–Day14), as depicted in Figure 2. Depression and anxiety increased significantly in the second period. Particularly, depression increased from 6.54 to 10.75% (*p* = 0.014), whereas anxiety increased from 3.69 to 6.49% (*p* = 0.040).

### Factors Associated With Health Outcomes

Table 3 shows the results of multivariable regression analyses used to examine the factors associated with each outcome of this study.

**Table 3:** Results of the multivariable regression analyses investigating factors associated with depression, anxiety, and HRQoL.

**Parameters**	**Depression**			**Anxiety**			**HRQOL**		
	**OR**	**95% CI**		**OR**	**95% CI**		**β**	**S.E**	**Pr > |t|**
Intercept	2.89	0.39	21.43	1.31	0.09	20.00	2.673	0.282	<0.0001
Quarantine period									
Second week	**1.78**	**1.10**	**2.88**	**4.18**	**1.44**	**12.09**	0.009	0.061	NS
(Ref = First week)									
Sex									
Female	**1.91**	**1.16**	**3.16**	1.31	0.69	2.50	**−0.174**	**0.061**	**0.0046**
(Ref = male)									
Age									
40 to 64	0.67	0.37	1.21	0.51	0.24	1.08	−0.008	0.077	NS
65 or over	0.48	0.05	4.49	0.87	0.10	7.95	0.082	0.219	NS
(Ref = 19 to 39)									
Education Level									
High school or less	1.22	0.68	2.21	0.90	0.40	2.03	−0.048	0.079	NS
(Ref = Tertiary education)									
Income									
Middle low	0.50	0.13	1.96	0.46	0.06	3.41	0.032	0.184	NS
Middle high	0.42	0.12	1.45	0.66	0.11	4.01	0.022	0.165	NS
High	0.48	0.14	1.67	0.46	0.07	2.86	0.047	0.163	NS
(Ref = Lowest)									
Marital status									
Single	1.06	0.60	1.88	0.53	0.26	1.10	−0.042	0.078	NS
Divorced/Widowed	1.42	0.50	4.04	0.10	0.01	1.67	−0.147	0.145	NS
(Ref = Married)									
Employment status									
Employer/Self-employed	0.99	0.38	2.56	0.98	0.26	3.67	−0.085	0.110	NS
Economically inactive	1.14	0.63	2.06	1.88	0.87	4.06	−0.006	0.078	NS
Others	1.82	0.85	3.87	**3.95**	**1.60**	**9.79**	−0.137	0.107	NS
(Ref = Salaried workers)									
Reason for quarantine									
Entrants from abroad	1.42	0.66	3.03	0.63	0.23	1.73	0.091	0.073	NS
(Ref = Contact tracers)									
Place to quarantine									
Other places	0.31	0.04	2.52	0.78	0.10	6.39	0.152	0.190	NS
Place of parents/relatives	1.10	0.51	2.34	2.02	0.83	4.92	−0.040	0.096	NS
(Ref = My home)									
Rationale for quarantine									
Correctly understood	**0.39**	**0.21**	**0.72**	0.51	0.23	1.14	0.177	0.105	0.0927
(Ref = No)									
Understanding of Instructions	**0.68**	**0.52**	**0.91**	0.72	0.51	1.03	**0.111**	**0.044**	**0.0109**
Perceived support	**0.74**	**0.55**	**0.99**	**0.66**	**0.45**	**0.96**	**0.074**	**0.031**	**0.0174**
No. of people staying together	1.08	0.90	1.30	1.05	0.83	1.33	−0.007	0.024	NS
Fear to infection
Yes (Ref = No)	1.56	0.38	6.49	**7.22**	**1.04**	**49.95**	0.006	0.064	NS
Self-rated Health state									
Bad/Very bad	1.97	0.80	4.84	2.00	0.44	8.96	**−0.562**	**0.142**	**<0.0001**
(Ref = Not bad)									
Predisposing Depression									
Yes (Ref = No)	**8.03**	**2.96**	**21.78**	3.29	0.60	18.07	**−0.914**	**0.304**	**0.0027**

*HRQoL was measured with EQ-5D index, Depression with PHQ-7, Anxiety with GAD-7. The bold values indicate statistically significant*.

### Depression

The logistic analysis showed that variables, such as quarantine period, sex, rationale for quarantine, the understanding of quarantine rules, perceived social support, and predisposing depression were all significantly associated with depression during the quarantine. Notably, the likelihood of suffering from depression in the second half of the quarantine period was 1.78 times (95% CI: 1.10–2.88) higher than that in the first half. As expected, women were more likely to suffer depression during quarantine than men (OR = 1.91 95% CI: 1.16–3.16), and those who already suffered depression as a predisposing health condition also displayed a higher likelihood to report depression during quarantine (OR = 8.03 95% CI: 2.96–21.78). However, people who were correctly aware of the rationale for quarantine (OR = 0.39 95% CI: 0.21–0.72) such as the need to protect others, those who understood the quarantine instructions well (OR = 0.68 95% CI: 0.52–0.91), and those who felt supported by others during self-quarantine (OR = 0.74 95% CI: 0.55–0.99) were all less likely to suffer from depression during quarantine.

### Anxiety

Anxiety was also significantly associated with the quarantine period, employment status, perceived support, and fear to infection. The second half of the 14-day quarantine period increased the likelihood of experiencing anxiety by a factor of 4.18 (95% CI: 1.44–12.09) when compared to the first half of the quarantine period. Those who reported their employment status as “others” were 3.95 times (95% CI: 1.60–9.79) more likely to develop anxiety than wage workers. Presumably, “others” implied they were, job-wise, in transition due to the pandemic. For instance, temporary workers who were expected to quit their jobs or were uncertain about their employment status. On the other hand, perceived support from others significantly reduced the likelihood of anxiety during quarantine (OR = 0.66, 95% CI: 0.45–0.96). Unlike depression, fear of further infection was the most critical factor in the manifestation of anxiety among quarantined individuals. People with a fear of being infected with COVID-19 tended to develop anxiety 7.22 times more than those who did not (95% CI: 1.04–49.95).

### HRQoL

A beta regression revealed no significant difference in HRQoL in the two halves of the quarantine period. Women had, on an average, 16.0% lower EQ-5D scores than men [exp (−0.174) = 0.84, *p* = 0.005]. A one-unit increase in the level of understanding of the quarantine rules significantly increased HRQoL by 11.7% (*p* = 0.0109). Moreover, consistent with the results of depression and anxiety, a one-unit increase level in perceived support from others during quarantine was associated with 7.7% higher EQ-5D scores (*p* = 0.0174). For those who considered themselves to have “bad” or “very bad” health conditions, the HRQoL scores reduced by 43.0% during quarantine when compared to others (moderate/good/very good) (*p* < 0.0001). Similar to depression, people with predisposing depression tended to experience a 59.9% decrease in EQ-5D scores than those without predisposing depression (*p* = 0.0027).

## Discussion

This online study is, to the best of our knowledge, the first attempt to investigate the significant factors contributing to depression, anxiety, and HRQoL among quarantined individuals living in Seoul, South Korea (*N* = 1,135) during the COVID-19 pandemic. According to our findings, factors including quarantine period, perceived social support, and knowledge about quarantine (such as the rationale for the quarantine and the quarantine rules) critically mattered for depression, anxiety, and quality of life of individuals under quarantine during the COVID-19 pandemic.

First, our study reconfirmed that the longer the quarantine period, the higher the likelihood of experiencing depression and anxiety. During the second half of the quarantine period, the likelihoods of depression and anxiety were 1.78 and 4.18 times higher than in the first half, respectively. This is consistent to findings outlined in previous studies (6, 8, 9, 32). Brooks et al. (6) identified the duration of quarantine as one of the stressors for poor mental health, and Hawryluck et al. (32) showed that more than 10 days of quarantine was highly associated with post-traumatic stress symptoms compared with those who underwent <10 days of quarantine. As a longer exposure to stressful situations can adversely affect one's mental health, long-term isolation can negatively affect mental health such as increased levels of depression and anxiety.

We also found that perceived support during quarantine was significantly associated with reduced incidence of depression and anxiety, as well as improved HRQoL. It is a well-known fact that social support is beneficial for mental and physical health (33–35). Although social support in this study was measured by a single-item questionnaire regarding the perceived levels of support by others during the quarantine, the findings comply to results from previous studies. However, our study is the first to reveal the significant association of social support with mental disorders and one's quality of life as experienced throughout quarantine amidst the COVID-19 pandemic.

Furthermore, it is noteworthy that a correct understanding behind the rationale for the need to quarantine, along with the related rules thereof, was one of the significant factors for successfully enduring self-quarantine without experiencing depression or with better quality of life overall. Brooks et al. (6) suggested that ensuring that the quarantined individuals have both a good understanding of the disease and the reason for quarantine by providing sufficient information should be prioritized because inadequate information acted as a stressor for those who had been quarantined. Reynolds et al. (36) have also suggested that the provision of a clear rationale to quarantined individuals, an improved preparation for the quarantine, or education thereof should be implemented to limit the psychological impact of the event, based on the experiences of the Severe Acute Respiratory Syndrome (SARS) outbreak.

Beyond these findings, there have been previous studies indicating that pre-existing mental health conditions are associated with increased risks of worsening mental health (9, 37, 38); this finding has been corroborated in our study. Even during the quarantine, those who have already suffered from depression were 8.03 times more likely to have depression and tended to have 59.9% reduced quality of life throughout quarantine. Thus, such cases must be specially cared for.

Furthermore, health authorities should pay attention to high-risk groups (e.g., people with pre-existing depression, limited social support, or women) and develop precautionary measures to prevent mental health disorders during quarantine. Education/communication with quarantined individuals to provide appropriate knowledge on quarantine and epidemiological information on the disease can help them become less stressed.

Studies on HRQoL during the COVID-19 quarantine have been inadequately addressed (15). Further studies are required regarding the health utility or disutility among those who are quarantined. We first investigated the factors influencing the EQ-5D scores of quarantined people in South Korea. However, research based on a nationwide sample is needed.

Additionally, our findings demonstrated that anxiety, but not depression or quality of life, was strongly associated with the fear of further infection and occupational stability. Self-rated health status only showed a significant association with HRQoL.

### Limitations

This study has some limitations that are important to note. First is the inherent age-based selection bias because the survey was online. Thus, older adults who are not comfortable with the use of the internet could easily be omitted. Additionally, people living in Seoul were the primary participants of this study. Hence, our findings are hardly generalizable to the entire population of South Korea. Second, the response rate was only 22.0% in our study. Although it is known that the typical downside of online survey is lower response rate (39), we conducted the online survey in consideration of the quarantining conditions of the study subjects. A variety of strategies including enticements in the form of incentives to complete surveys is recommended to improve the response rate of the online survey (39). However, we didn't provide any form of incentives to increase the response rate in this study. Third, one of our major findings was that predisposing depression was 8.03 times more likely to develop depression during COVID 19 quarantine. However, only 25 out of 1,135 (2.2%) reported having suffered from depression prior to quarantine, which may incorporate sampling error. Further studies with larger populations are needed. Lastly, we had to slightly modify the timeframes of the validated measures of depression (PHQ-9) and anxiety (GAD-7) to suit the limited setting of the quarantine.

### Recommendations

With the prolonged combat against COVID-19, healthy quarantine is required. Particularly, high-risk groups (e.g., people with pre-existing depression, limited social support, or women) should be provided with special attention and undertake precautionary prevention measures. Various measures should be taken to actively support the quarantined people at the community level, as led by the district office. Furthermore, a clear explanation of the quarantine guidelines seems to be critical for those who are quarantined. Since the degree of understanding of the guidelines and the purpose of quarantine proved significant in improving the quality of life and preventing depressive symptoms, it is important to educate and communicate with people in quarantine. As it was found that longer periods of quarantine were associated with increased incidence of depression and anxiety, precautionary measures should be developed in accordance with the quarantine period and appropriately implemented to prevent depression and anxiety.

## Conclusions

This study highlights the need to develop precautionary measures to prevent depression and anxiety among people undergoing COVID-19 quarantine. In particular, individuals with depression prior to quarantine should be carefully monitored during the quarantine. Further studies with larger populations are needed.

## Data Availability Statement

The raw data supporting the conclusions of this article will be made available by the authors, without undue reservation.

## Ethics Statement

The Institutional Review board of Seoul Metropolitan City (IRB No. 2020-10-0001) approved this study. All participants provided online informed consent before beginning the survey. The patients/participants provided their written informed consent to participate in this study.

## Author Contributions

H-YK conceived the study. H-YK, YK, and S-YL established the consortium between Seoul Health Foundation and the affiliated districts of the Seoul Metropolitan Government. H-YK and S-YL performed the analyses. H-YK and YK drafted the manuscript. All authors contributed to finalizing the manuscript.

## Conflict of Interest

The authors declare that the research was conducted in the absence of any commercial or financial relationships that could be construed as a potential conflict of interest.

## Publisher's Note

All claims expressed in this article are solely those of the authors and do not necessarily represent those of their affiliated organizations, or those of the publisher, the editors and the reviewers. Any product that may be evaluated in this article, or claim that may be made by its manufacturer, is not guaranteed or endorsed by the publisher.

## Abbreviations

COVID-19: SARS-CoV-2 virus diseases 2019
PHQ-9: Patient Health Questionnaires-9
GAD-7: Generalized Anxiety Disorders-7
HRQoL: Health-related Quality of Life
EQ-5D: Euroqol-5 Dimensions.
